# Serial Amnioinfusion Therapy for Treatment of Congenital Bilateral Renal Agenesis—A Systematic Review

**DOI:** 10.1002/pd.6850

**Published:** 2025-07-18

**Authors:** Adriana Baez, Gabriele Tonni, Chryso P. Katsoufis, Amanda Alladin, Ugo Maria Pierucci, Yair J. Blumenfeld, Rodrigo Ruano

**Affiliations:** ^1^ Miller School of Medicine University of Miami Miami Florida USA; ^2^ Department of Obstetrics & Neonatology, and, Researcher Istituto di Ricovero e Cura a Carattere Scientifico (IRCCS) Azienda USL Reggio Emilia Reggio Emilia Italy; ^3^ Division of Pediatric Nephrology Department of Pediatrics University of Miami Miller School of Medicine Miami Florida USA; ^4^ Division of Pediatric Critical Care Department of Pediatrics University of Miami Miller School of Medicine Miami Florida USA; ^5^ Department of Pediatric Surgery “V. Buzzi” Children’s Hospital Milan Italy; ^6^ Division of Maternal‐Fetal Medicine and Obstetrics Department of Obstetrics & Gynecology Stanford University School of Medicine Stanford California USA; ^7^ Division of Maternal‐Fetal Medicine Department of Obstetrics, Gynecology & Reproductive Sciences University of Miami Miller School of Medicine Miami Florida USA

## Abstract

Serial amnioinfusion therapy (SAT) has emerged as a potential mitigatory intervention to adverse perinatal outcomes associated with congenital bilateral renal agenesis (BRA). However, its efficacy, safety, and ethical implications warrant thorough evaluation. This systematic review, developed according to PRISMA guidelines, analyzes the published data on outcomes of SAT for BRA and explores its implications. Inclusion criteria were a diagnosis of bilateral renal agenesis, therapeutic use of amnioinfusion, amnioinfusion procedure, and individual maternal and fetal outcome reports. A total of 192 published studies were identified. Among these, 11 full texts were included (*N* = 40). Only cases resulting in live birth and with reported maternal and neonatal outcomes were analyzed. The average number of amnioinfusions per mother was 9 (*n* = 23; range 1–26 infusions). Median gestational age at delivery was 33.4 weeks (*n* = 40; range 23.7–36.8 weeks). APGAR scores (*n* = 14) at 1 and 5 min were 4 and 6, respectively. Almost half of newborns died within 33 days of life (*n* = 19) and 7 (17.5%) survived at the time of original publication. Overall neonatal mortality was 82.5% (33 of 40). These findings suggest that SAT for BRA improves the chances of neonatal survival in the first few days to weeks of life but not consistently beyond that time. Additional advances in neonatal care are needed to improve long‐term outcomes in peripartum survivors.

## Summary

- What's already known about this topic?
  ◦ Serial amnioinfusion therapy (SAT) is used to mitigate pulmonary hypoplasia (PH) in fetuses with bilateral renal agenesis (BRA).
  ◦ Perinatal mortality remains high, and survivors typically require dialysis and eventual transplantation.
  ◦ No standardized guidelines currently exist for SAT in this population.
  ◦ The existing literature consists primarily of small‐scale case reports and observational studies.
- What does this study add?
  ◦ This is the most comprehensive systematic review to date focused specifically on SAT in BRA, including 40 cases from 11 full‐text studies.
  ◦ While SAT may improve short‐term neonatal survival, long‐term survival and transplant outcomes remain rare.
  ◦ The review emphasizes significant maternal and neonatal risks, the absence of standardized protocols, and the need for improved postnatal care pathways.
  ◦ These findings support the need for multicenter registries and caution in offering SAT outside of investigational contexts.

## 1 Introduction

Bilateral renal agenesis (BRA) is a rare congenital anomaly long considered uniformly fatal due to the resulting anhydramnios and consequent pulmonary hypoplasia (PH) [1, 2, 3, 4, 5]. According to a recent population‐based registry, BRA occurs in approximately 1 in 3000 pregnancies and may arise from both genetic and non‐genetic etiologies. Advances in prenatal molecular diagnostics have expanded our understanding of monogenic causes [1]. From the second trimester onward, fetal urine contributes over 90% of the amniotic fluid volume, supporting fetal lung expansion and development [2, 3, 4]. In the absence of renal function, anhydramnios impairs thoracic growth, and the resulting PH is typically incompatible with postnatal life [5].

Although postnatal kidney replacement therapy (KRT) has significantly improved survival in infants with end‐stage kidney disease from other causes, its application in BRA is limited by the severity of PH and the challenges of neonatal respiratory support [6]. Considering this, BRA has been proposed as a potential target for prenatal intervention despite the absence of established clinical guidelines [4, 7, 8].

Serial amnioinfusion therapy (SAT) was introduced in the 1980s to restore intrauterine fluid levels, aiming to alleviate fetal thoracic compression and promote lung development [9, 10, 11]. Although SAT may mitigate PH, it is associated with maternal‐fetal risks such as infection, preterm delivery, and PPROM [3, 6, 7]. Evidence supporting its use remains limited and heterogeneous, consisting largely of retrospective reports and small case series. The recent publication of the renal anhydramnios fetal therapy (RAFT) trial, the first prospective multicenter study of SAT in BRA, offers important insights into its feasibility and neonatal outcomes [3].

In the absence of a standardized approach, a systematic review of the available literature is warranted. This study aims to synthesize reported outcomes of SAT in fetuses with BRA, assess associated maternal and neonatal risks, and identify ethical and clinical considerations relevant to future research and counseling.

## 2 Methods

### 2.1 Literature Search

**FIGURE 1:**
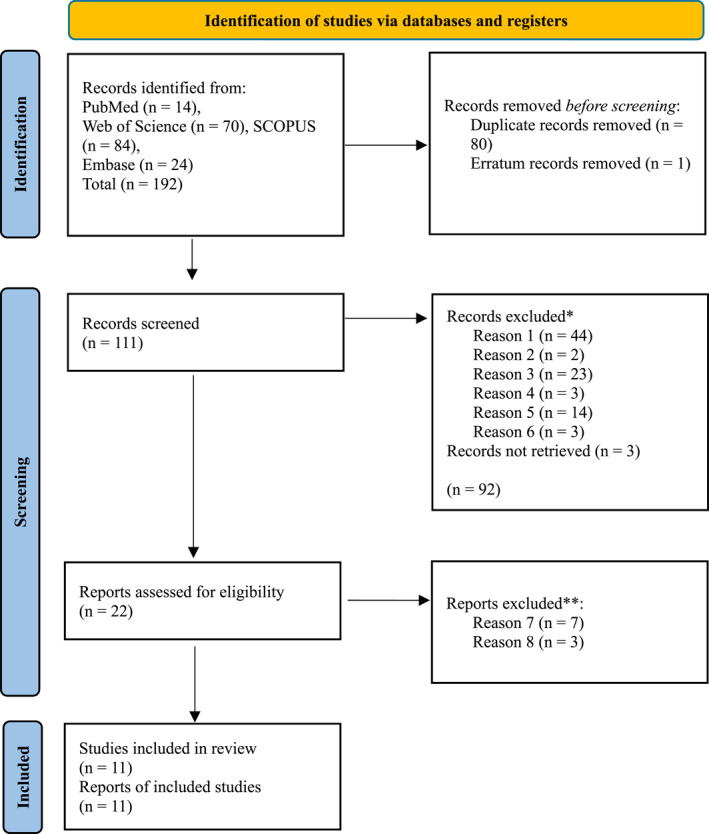
Literature search flow diagram with keywords including *serial amnioinfusion therapy* and *renal agenesis*. *Reason 1: No diagnosis of BRA, Reason 2: Language other than English, Reason 3: No intervention, Reason 4: Commentary article, Reason 5: Wrong indication for amnioinfusion, Reason 6: Wrong therapy. **Reason 7: Not enough detail or no individual patient data, Reason 8: No full text access.

A comprehensive systematic review protocol was developed for this study, according to Preferred Reporting Items for Systematic reviews and Meta‐Analyses (PRISMA) guidelines. A search was conducted across PubMed/Medline, EMBASE, Web of Science (WOS) and SCOPUS for articles published from the date of database inception to January 2025 for publications including the search terms of “renal agenesis” and “serial amnioinfusion therapy”. There were no restrictions to language or manuscript type in the initial search (Figure 1). This study was registered at PROSPERO (CRD42025634007).

### 2.2 Screening and Selection

Once records were identified, duplicates and *erratum* records were excluded. The primary author (AB) then screened the abstracts of the remaining records according to the established eligibility criteria based on the population, intervention, comparison, outcome (PICO) framework. Full‐text articles were then reviewed by the primary author to make the final inclusion decisions. Records were subjected to the following inclusion criteria: a diagnosis of BRA in at least one patient, the use of amnioinfusion therapy to improve maternal and fetal outcomes, amnioinfusion therapy procedure, maternal outcome reports, and fetal outcome reports. Exclusion criteria included studies that did not sufficiently report treatment and outcome values for individual participants. Studies with patients receiving amnioinfusion for other diagnoses were excluded if the BRA patient data could not be parsed from the larger study cohort. Risk of bias was assessed using the Newcastle‐Ottawa scale (NOS), a tool designed to evaluate the quality of non‐randomized studies [12]. The primary author and the corresponding author (RR) independently performed the NOS analysis to ensure consistency and rigor in evaluating the included studies.

### 2.3 Data Abstraction

Variables used for data abstraction include authors, title of the article, year of publication, maternal age, number of amnioinfusions, gestational age (GA) at the start of infusion therapy, infusion technique, procedure complications, GA at delivery, birth weight, APGAR score at 1 and 5 min, pregnancy or delivery complications, neonatal complications, dialysis duration, dialysis modality, kidney transplant, survival outcome, and age at death (if applicable). Only cases that resulted in live birth and reported both maternal and neonatal outcomes were eligible for data abstraction as postnatal survival and intervention data were central to the review’s objectives. Data were obtained from the abstract, main text, figures, tables. Data were then analyzed with IBM SPSS Statistics Version 28.0.2.0. Due to the necessary inclusion of different types of studies, there was significant variability across the publications, making it challenging to compare them directly. Categorical variables were analyzed as frequencies and percentages, and continuous variables by mean or median values, where indicated. Although different techniques were employed across cases (percutaneous serial amnioinfusions vs. amnioport), all were included given their common therapeutic intent. However, due to the limited sample size and lack of consistent data, comparative subgroup analysis by technique was not feasible, and this heterogeneity may impact the generalizability of our findings.

## 3 Results

One hundred ninety‐two cases were identified from the literature search (Figure 1). With duplicate and *erratum* cases removed (*n* = 81), 111 records were screened for eligibility. 94 studies were excluded for ineligibility due to patient demographics, amnioinfusions as a diagnostic technique (rather than therapeutic), study of physiology as opposed to therapy, and lack of intervention management. After initial abstract screening and full text retrieval, 17 publications were analyzed according to the inclusion and exclusion criteria. Among these, 11 studies met the criteria of appropriate intervention and extractable individual subject data, resulting in 40 eligible participants. See Table 1 for data on participant inclusion by paper, as well as the risk of bias score.

**TABLE 1:** Participant inclusion by author.

Study	Number of participants	%	Type of study	Risk of bias (ROB)
Cameron et al. [13]	1	2.5	Case study	4/9 (moderate to high)
Riddle, Habli et al. [14]	8	20	Institutional review board‐approved retrospective review	7/9 (moderate)
Whittaker and Leonardi [15]	1	2.5	Conference abstract	4/9 (moderate to high)
Miller et al. [3]	17	42.5	Prospective, nonrandomized clinical trial	7/9 (moderate)
Ogundipe et al. [16]	5	12.5	Retrospective review	5/9 (moderate to high)
Warring et al. [17]	1	2.5	Systematic review	6/9 (moderate)
Bienstock et al. [18]	1	2.5	Case study	5/9 (moderate to high)
Hsu et al. [11]	2	5	Retrospective study	5/9 (moderate to high)
Sheldon et al. [19]	1	2.5	Case series	5/9 (moderate to high)
S. L. Riddle et al. [20]	2	5	Case series	5/9 (moderate to high)
Polzin et al. [21]	1	2.5	Methods article	5/9 (moderate to high)
Total	40	100		

Abbreviation: % = percent.

Cases were from the United States (*n* = 37), Taiwan (*n* = 2), and Canada (*n* = 1), and publication dates ranged from 1994 to 2023. See demographic data for all patients in Table 2. All included texts provided information on amnioinfusion technique, GA at delivery, post‐delivery complications, dialysis duration, and survival outcome. Studies differed in disclosed data related to maternal age, total fluid per amnioinfusion, type of delivery, birth weight, and APGAR scores.

**TABLE 2:** Descriptive statistics of participants.

Total participants *N* = 40		Maternal age	GA at start of SAT (wk)	GA at delivery (wk)	Birth weight (g)	APGAR 1 min	APGAR 5 min	Age at time of death (d)
*N*	Data available	12	20	40	26	14	14	33
	Missing	28	20	0	14	26	26	7
Mean		—	—	—	—	3.79	—	—
Median		27.50	23.357	33.414	1952.50	—	6.50	23.00
Minimum		21	16.0	23.7	460	0	0	1
Maximum		39	34.9	36.9	2700	8	9	760
%‐ile	25	25.00	21.071	30.250	1475.00	1.00	3.25	1.00
	50	27.50	23.357	33.414	1952.50	4.00	6.50	23.00
	75	33.00	26.000	35.000	2125.00	7.00	8.00	121.00

*Note:* Data normally distributed, determined by a skew less than ± 0.5 is described by the value of the mean, whereas data in violation of normal distribution is represented by the median value.

Abbreviations: d = days, g = grams, GA = gestational age, min = minutes, SAT = serial amnioinfusion therapy, wk = weeks.

The median age of women who underwent SAT was 27.5 years (*n* = 12; range 21–39 years). The median number of amnioinfusions per pregnancy was 9 (*n* = 23; range 1–26 infusions), with techniques including percutaneous (*n* = 36), amnioport (*n* = 3), and combined percutaneous and amnioport (*n* = 1) infusions. Median GA at the start of infusions was 24.1 weeks (*n* = 20; range 16–34.9 weeks). Complications of SAT included difficulty placing the needle, persistent fetal bradycardia, amnioport malfunction, PPROM, fluid infused into the subcutaneous space, chorioamniotic separation, migration of port tubing, chorioamnionitis, premature rupture of membranes (PROM) with resealing, positive amniotic fluid culture, amnion‐chorion separation, anhydramnios within 48 h of infusion (due to intramembranous/subcutaneous/placental absorption), and preterm labor. Descriptive data for all patients on SAT technique, mode of dialysis, outcome status, and transplant status are included in Table 3.

**TABLE 3:** Descriptive data of SAT interval, dialysis interval, and outcome.

		Number of participants	%
Amnioinfusion technique	Percutaneous	36	90
	Combined percutaneous and amnioport	3	7.5
	Amnioport	1	2.5
Mode of dialysis	Peritoneal dialysis	26	65
	Hemodialysis	7	17.5
	CKRT	3	7.5
	Aquapheresis	9	22.5
	Patients requiring 2 or more modalities	17	42.5
Status	Deceased	33	82.5
	Alive	7	17.5
Transplant received?	No	37	92.5
	Yes	3	7.5

Abbreviations: % = percent, CK = continuous kidney replacement therapy.

GA at the time of delivery was 32.5 weeks (*n* = 40; range 23.7–36.8 weeks). APGAR scores (*n* = 14) at 1 and 5 min were 4 (range 0–8) and 6 (range 0–9), respectively. The mode of delivery was more commonly by cesarean section (*n* = 11, 57.9%) versus vaginal delivery (*n* = 8, 42.1%). A detailed list of peri‐partum maternal complications is provided in Table 4, and neonatal complications after delivery by organ system are provided in Table 5.

**TABLE 4:** Maternal complications following SAT.

Complication	Number of mothers	%
Difficulty or discomfort with needle placement	1	2.5
Incorrect infusion technique (infused in to subcutaneous space)	2	5
Chorioamniotic separation	5	12.5
Port malfunction	2	5
PROM/PPROM	7	17.5
Chorioamnionitis	3	7.5

Abbreviations: % = percent, PPROM = prelabor premature rupture of membrane, PROM = premature rupture of membranes.

**TABLE 5:** Post‐delivery neonatal complications by organ system.

Complication	*N*	%
Respiratory		
Early/prolonged intubation or resuscitation	7	17.5
Pneumothorax	6	15
Pulmonary interstitial edema	1	2.5
Pulmonary hypertension	2	5
Respiratory failure resulting in death at delivery	3	7.5
Pulmonary hemorrhage	3	7.5
Adenovirus‐associated respiratory failure	1	2.5
Gastrointestinal		
Pneumoperitoneum	1	2.5
Necrotizing enterocolitis ± intestinal perforation	3	7.5
Cardiovascular		
Hypotension	15	37.5
Cardiac arrest	6	16
Cardiac arrhythmias	1	2.5
Neurological		
Ischemic brain injury	2	5
Seizures	1	2.5
Stroke	5	12.5
Brain hemorrhage	2	5
General		
Sepsis	8	20
Multiple organ system failure	2	5
Dialysis‐related		
Persistent peritoneal dialysis catheter leakage or obstruction	4	10
Peritonitis secondary to dialysis	8	20

Abbreviation: % = percent.

Of the 40 patients' neonates who survived to delivery, 33 (82.5%) demised and 7 (17.5%) were alive at the time of original manuscript publication. Table 6 compares maternal treatment interval data and short‐term outcome data by outcome status at the time of publication. According to an independent samples *t*‐test, alive versus deceased groups differed significantly by maternal age (*t*[10] = −5.37, *p* < 0.001), but not by number of amnioinfusions (*t*[21] = 0.65, *p* = 0.53), GA at the start of amnioinfusions (*t*[18] = −0.12, *p* = 0.91), delivery GA (*t*[38] = −0.42, *p* = 0.68), or birth weight (*t*[24] = −0.37, *p* = 0.72). According to a Mann‐Whitney test, groups did not differ significantly by APGAR at minute 1 (*U* = 6.00, *Z* = −1.66, *p* = 0.126, Exact Sig.) or minute 5 (*U* = 8.00, *Z* = −1.34, *p* = 0.225, Exact Sig.). Of the non‐survivors, half died within 33 days of life (*n* = 19). Of the 7 survivors at the time of publication, 3 had received a kidney transplant, including the patient described in the Bienstock et al. [18] in 2014. Table 7 displays the relevant survival interval for patients alive at the original manuscript publication. Although we initially considered stratifying outcomes by GA at the initiation of SAT, the distribution of cases did not support meaningful statistical analysis. Only two cases began SAT after 30 weeks' gestation, limiting the interpretability of stratification above versus below a given GA threshold. As such, we report GA at SAT initiation for each survivor in Table 7.

**TABLE 6:** Comparison of treatment interval and birth data by living status.

	Living status	Number of participants	Mean
Maternal age (yr)*	Deceased	9	26^a^
	Alive	3	36.00
Number of amnioinfusions	Deceased	20	11.20
	Alive	3	8.00
GA at start of amnioinfusions (wk)	Deceased	17	24.05
	Alive	3	24.43
Delivery GA (wk)	Deceased	33	32.42
	Alive	7	32.98
Birth weight (g)	Deceased	19	1802.42
	Alive	7	1940^a^
Apgar 1	Deceased	11	3.09
	Alive	3	6.33
Apgar 5	Deceased	11	5.00
	Alive	3	7.67

Abbreviations: g = grams, GA = gestational age, wk = weeks, yr = years.

^a^ Median value reported, as skew is less than ± 0.5.

* Statistically significant, *p* < 0.001.

**TABLE 7:** Summary of therapy course and outcome for survivors.

Transplant received?	No	No	No	No	No	Yes	Yes
Age at publication	23 mths	9 mths	15 mths	24 mths	4 yrs	54 mths	44 mths
Neonatal complications	Hypotension, seizures, left posterior stroke	Hypotension, supraventricular tachycardia, grade 1 germinal matrix hemorrhage, seizures	Hypotension	Left posterior stroke	Emergent resuscitation, intubation, pneumoperitoneum, cardiopulmonary arrest secondary to massive blood loss	Obstruction of PD catheter complicated by peritonitis, prolonged hospitalization	Right pneumothorax, pulmonary interstitial edema
APGAR 1, 5 min	NR	NR	NR	NR	8, 9	7, 8	4, 5
Birth weight (g)	1500	1800	2030	2700	2000	1230	1940
GA at delivery (wk + d)	33 + 0	33 + 0	33 + 0	34 + 0	35 + 0	28 + 5	34 + 1
GA at start of SAT (wk + d)	24 + 0	24 + 5	24 + 5	24	23 + 5	27 + 0	26 + 5
SAT complications	NR	NR	NR	NR	Amnion‐chorion separation, anhydramnios	NR	PPROM
Total fluid per amnioinfusion	300–800 mL	300–800 mL	300–800 mL	300–800 mL	200–410 mL	∼15 mL per week of gestational age	NR
Participant *N*.	24	25	26	27	28	34	37

Abbreviations: GA = gestational age, min = minute, Mths = month, No. = number, NR = not reported, PD = peritoneal dialysis, yrs = years.

To better represent variability in follow‐up duration and to provide clarity on the survival trajectory of all subjects, we have included a summary table (Table 8) that presents outcomes at the study level, including number of live births, survivors at publication, reported deaths, transplant status, and relevant outcome notes.

**TABLE 8:** Summary of included studies evaluating serial amnioinfusion therapy (SAT) for bilateral renal agenesis (BRA).

Study	Participants (*n*)	Live births	Survivors at publication	Deaths reported	Kidney transplants	Key notes
Cameron et al. [13]	1	1	0	1	0	First SAT case reported; death at 23 days due to dialysis failure
Riddle, Habli et al. [14]	8	8	0	8	0	Sepsis‐related deaths; no survivors to discharge
Whittaker and Leonardi [15]	1	1	0	1	0	Abstract only; survival to 5 months
Miller et al. [3]	17	17	7	10	0	Largest cohort; 6 survived to discharge, 4 remained alive long‐term
Ogundipe et al. [16]	5	5	1	4	0	One 4‐year survivor; others died from dialysis failure or sepsis
Warring et al. [17]	1	1	1	0	0	Mixed cohort: One case survived to 7 months
Bienstock et al. [18]	1	1	1	0	1	Survived to 9 months; underwent successful kidney transplant.
Hsu et al. [11]	2	2	1	1	0	Early experience; limited neonatal follow‐up
Sheldon et al. [19]	2	2	2	0	2	Two cases of successful kidney transplant following SAT
S.L. Riddle et al. [20]	2	2	0	2	0	Neonatal deaths from refractory hypotension
Polzin et al. [21]	1	1	1	0	0	Successful amnioport use; no major complications reported

Abbreviations: BRA = bilateral renal agenesis, PD = peritoneal dialysis, RAFT = renal anhydramnios fetal therapy trial, SAT = serial amnioinfusion therapy.

## 4 Discussion

This systematic review synthesizes data from 11 published studies, encompassing a total of 40 fetuses diagnosed with BRA who underwent SAT. Our analysis supports the finding that SAT may mitigate the immediate lethality of PH in a subset of cases, enabling survival into the neonatal period. Of the 40 liveborn infants, 7 (17.5%) were reported alive at the time of original publication, and only 3 had received kidney transplantation [3, 18, 19]. The overall neonatal mortality rate was 82.5%, primarily due to dialysis failure, severe respiratory compromise, or sepsis. These findings highlight both the clinical potential and the limitations of SAT in addressing the perinatal lethality associated with BRA.

This review highlights the lack of consensus regarding the technical parameters of SAT. The gestational age at initiation of therapy ranged from 16.0 to 34.9 weeks, the number of infusions varied widely (range: 1–26), and infusion techniques included percutaneous, amnioport‐based, and combined approaches [3, 16, 17, 22]. Moreover, infusion volumes, intervals between procedures, and monitoring protocols were inconsistently reported. Despite the common aim to restore intrauterine amniotic fluid volume and promote fetal lung development, no standardized protocol has been adopted, and the literature is marked by significant procedural variability.

Recommendations in the literature are discordant. Fisk et al. suggest careful pressure monitoring during SAT to avoid overdistension and PROM [23], while Stefos et al. advocate for fewer, high‐volume infusions to minimize procedural frequency and cumulative risk [24]. The absence of standard criteria to assess treatment success, whether via pulmonary metrics, gestational age at delivery, or neonatal oxygenation, further complicates the evaluation of efficacy.

Historical case reports reflect the ongoing uncertainty surrounding SAT. The first documented case, reported by Cameron et al. in 1994, demonstrated transient respiratory improvement but ultimately resulted in neonatal death due to failure of PD and severe neurological injury [13]. Over 2 decades later, a systematic review by Warring et al. identified only one surviving infant (at 9 months of age) out of eight fetuses with BRA treated with SAT, and noted that most neonates either died shortly after birth or never reached viability [17]. Their analysis also introduced a candidate selection framework based on fetal growth, GA, and the presence of comorbidities, parameters that remain clinically relevant but are rarely applied in a standardized fashion.

The renal anhydramnios fetal therapy (RAFT) trial, published in 2023, represents the first multicenter prospective investigation of SAT in BRA using a predefined treatment protocol [3]. Among 17 liveborn infants in the BRA arm, 14 (82%) survived to day 14 postnatally. However, only 6 (35%) were discharged from the hospital on dialysis, and none had received a kidney transplant at the time of reporting. These data highlight that while SAT may prevent early neonatal death by restoring fluid volume and supporting lung development, its impact on long‐term survival remains limited. Many infants experienced complications necessitating multiple dialysis modalities, often due to body size, catheter failure, or hemodynamic instability [25, 26]. The delay in reaching transplant eligibility was frequently attributed to growth restriction and the presence of lower urinary tract anomalies, conditions which often coexist with BRA [3].

### 4.1 Maternal Risk and Procedural Burden

SAT involves repeated intrauterine procedures and is associated with significant maternal risks. In the RAFT trial, preterm premature rupture of membranes (PPROM) occurred in 61% of participants, chorioamniotic separation in 28%, and vaginal bleeding in 22% [3]. A larger retrospective study by Riddle et al. reported complications in 75% of mothers undergoing SAT, including chorioamnionitis and spontaneous preterm labor [22]. While some groups have explored the use of subcutaneous amnioport systems as a less invasive delivery method for fluid supplementation, concerns about infection, port malfunction, and rejection persist [27]. Importantly, no maternal deaths were reported in any included study.

### 4.2 Neonatal Outcomes and Resource Requirements

Even in cases where SAT enables live birth, intensive neonatal care is almost universally required. Infants typically require immediate intubation, prolonged mechanical ventilation, and complex fluid and electrolyte management. In our review, all survivors experienced at least one severe complication, including ischemic brain injury, sepsis, necrotizing enterocolitis, and multiorgan dysfunction [3, 19]. Only three infants across all reports received a kidney transplant, and both did so months after birth following lengthy hospitalizations and home dialysis support [18, 19].

The resource demands for managing these infants are considerable. Many require more than 60 days of inpatient care, multiple surgical procedures, and access to multidisciplinary neonatology, nephrology, surgery, and social work support teams. Given the low incidence of pediatric end‐stage renal disease (ESRD), these resources are concentrated in a small number of specialized centers, raising concerns about equitable access [28, 29, 30, 31, 32].

### 4.3 Ethical Considerations and the Role of Informed Consent

SAT in the context of BRA poses complex ethical challenges. While the procedure may extend life in an otherwise fatal condition, it frequently results in prolonged suffering and uncertain quality of life. Parents may perceive SAT as a hopeful intervention, but consent must be based on a realistic understanding of neonatal prognosis, transplant candidacy, and the long‐term burden of chronic illness [33, 34, 35]. The importance of structured counseling, early palliative care involvement, and multidisciplinary support cannot be overstated.

In our analysis, even the most optimistic survival outcome (17.5%) should be viewed in light of extensive resource needs and the absence of long‐term data on quality of life. For families choosing to pursue therapy, shared decision‐making supported by ethics consultation is essential.

### 4.4 Limitations

This review is limited by the overall low quality of the included data. More than half of the cases were derived from retrospective case reports or case series, and one was based on a conference abstract without full methodological details [13, 15, 17]. The heterogeneity in data collection, absence of standardized outcome measures, and variability in follow‐up durations introduce bias and limit the generalizability of our findings. The risk of publication bias must also be considered, as successful or exceptional cases are more likely to be published, potentially inflating perceptions of benefit.

In addition, our ability to analyze the influence of gestational age at SAT initiation was limited. While we hypothesized that earlier initiation might improve outcomes, only two cases in our review involved initiation at or after 30 weeks' gestation, precluding meaningful statistical stratification. These cases were discussed narratively, and GA at initiation was reported for all survivors. Future research should explore the prognostic relevance of earlier SAT initiation using larger and prospectively collected datasets.

Moreover, neonatal care practices, including dialysis modalities, catheter types, and thresholds for initiating or withdrawing support, differ across institutions and likely influence outcomes. Our review could not fully account for these center‐level variables.

## 5 Conclusion

This systematic review suggests that SAT may reduce the immediate lethality associated with pulmonary hypoplasia in fetuses with BRA, enabling short‐term postnatal survival in a subset of cases. However, SAT remains a high‐risk intervention with uncertain long‐term benefit, as most survivors face significant neonatal morbidity, and transplant success is rare.

While SAT shows promise as a regenerative strategy to restore amniotic fluid volume and support fetal lung development, its overall impact on long‐term survival and quality of life is limited. The data highlight the importance of early, specialized postnatal care, particularly access to neonatal dialysis and multidisciplinary management, as a prerequisite to meaningful outcomes.

For families choosing to continue a pregnancy affected by BRA, psychosocial, financial, and logistical burdens remain substantial, and ethical considerations must be addressed with transparency and support. Future studies involving larger, prospective cohorts are needed not only to clarify the potential role of SAT, but also to optimize neonatal care pathways and assess which patients might benefit most. Continued evaluation of the RAFT trial and the development of international registries will be essential to guide practice.

## Ethics Statement

The authors have nothing to report.

## Consent

The authors have nothing to report.

## Conflicts of Interest

The authors declare no conflicts of interest.

## Supporting information

Supporting Information S1

## Data Availability

No new data were created or analyzed in this study. Data sharing is not applicable to this article.
